# Physisorbed Polymer-Tethered Lipid Bilayer with Lipopolymer Gradient

**DOI:** 10.3390/ma5112243

**Published:** 2012-11-08

**Authors:** Yu-Hung Lin, Daniel E. Minner, Vincent L. Herring, Christoph A. Naumann

**Affiliations:** 1 Department of Chemistry and Chemical Biology, Indiana University-Purdue University Indianapolis, 402 N Blackford St., Indianapolis, IN 46202, USA; E-Mails: yuhulin@iupui.edu (Y.-H.L.); dminner@iupui.edu (D.E.M.); vherring@iupui.edu (V.L.H.); 2 Integrated Nanosystems Development Institute, Indiana University-Purdue University Indianapolis, 755 W Michigan St., Indianapolis, IN 46202, USA

**Keywords:** gradient bilayer, Langmuir-Blodgett transfer, lipopolymer, polymer-tethered lipid membrane, Epifluorescence, atomic force microscopy, buckling structure

## Abstract

Physisorbed polymer-tethered lipid bilayers consisting of phospholipids and lipopolymers represent an attractive planar model membrane platform, in which bilayer fluidity and membrane elastic properties can be regulated through lipopolymer molar concentration. Herein we report a method for the fabrication of such a planar model membrane system with a lateral gradient of lipopolymer density. In addition, a procedure is described, which leads to a sharp boundary between regions of low and high lipopolymer molar concentrations. Resulting gradients and sharp boundaries are visualized on the basis of membrane buckling structures at elevated lipopolymer concentrations using epifluorescence microscopy and atomic force microscopy. Furthermore, results from spot photobleaching experiments are presented, which provide insight into the lipid lateral fluidity in these model membrane architectures. The presented experimental data highlight a planar, solid-supported membrane characterized by fascinating length scale-dependent dynamics and elastic properties with remarkable parallels to those observed in cellular membranes.

## 1. Introduction

The surface functionalization of solid and polymeric materials with biomembrane-mimicking supramolecular assemblies has fascinated research groups for nearly three decades. Such model membrane architectures represent an intriguing interface between the biological world and a broad range of highly sensitive biophysical detection techniques, and have potential significance in different biotechnological applications [1]. Moreover, they enable the characterization of biomembrane properties under well-defined conditions. The solid-supported phospholipid bilayer represents one of the simplest types of biomembrane-mimicking supramolecular assemblies on a solid substrate. Its thermodynamic properties (e.g., lipid mixing and phase transition behavior) are similar to those of a free lipid bilayer. Due to the lubrication effect of the thin water layer between bilayer and hydrophilic substrate, solid-supported lipid bilayer systems can exhibit substantial long-range lateral mobility, thus mimicking the functionally important membrane fluidity found in biological membranes. Since Tamm and McConnell first reported the fabrication of a solid-supported lipid bilayer using two successive monolayer transfers in 1985 [2], this type of model membrane system has been successfully utilized in a wide range of exciting research applications. Prominent examples include the characterization of processes associated with T-cell signaling [3,4], vesicle adhesion [5], and biosensor applications [6]. However, the close proximity between bilayer and solid substrate can induce substrate-induced artifacts that limit the applicability of solid-supported lipid bilayers for studies involving transmembrane proteins [7]. To overcome this limitation, polymer-supported lipid bilayers were introduced, in which the hydrophilic polymer layer between bilayer and solid substrate leads to a controlled uplift of the bilayer from the underlying substrate [8,9]. Stable polymer-supported lipid bilayers can be built by means of covalent tethering [10] or attractive electrostatic interactions [11] at the polymer-bilayer interface. They can be either chemisorbed [12,13,14] or physisorbed [15] to the underlying substrate.

**Figure 1:**
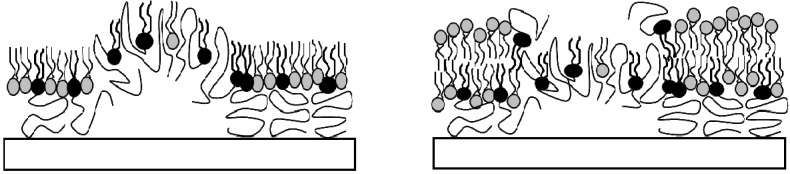
Schematic of stress relaxation processes in physisorbed polymer-tethered lipid monolayer (left) and bilayer (right). Buckling delamination of the monolayer is caused by elevated concentrations of membrane stress-inducing lipopolymers. Penetration of polymer chains into the hydrophobic region of the buckled monolayer prevents formation of the bilayer at buckling structures [23].

Polymer-tethered lipid bilayer systems comprised of phospholipids and lipopolymers represent one particularly attractive type of polymer-supported membrane. Their main advantage lies in the straightforward control of tethering density by pre-organizing a mixed lipid-lipopolymer monolayer at the air-water interface and subsequently transferring it to the solid substrate using Langmuir-Blodgett (LB) deposition, followed by bilayer completion via vesicle fusion [12] or Langmuir-Schaefer (LS) transfer [13]. These membrane systems have been successfully employed to investigate properties of reconstituted transmembrane proteins, which maintained notable lateral mobility [12,15,16,17]. The polymer-mediated reduction of substrate-bilayer interactions has also been used to explore the transbilayer coupling of raft-like domains in ternary, raft-mimicking lipid mixtures [18,19]. Both types of experiments are typically conducted using low tethering concentrations. However, an interesting feature of lipopolymer-containing polymer-tethered membranes lies in the ability to change membrane dynamics, organization, and elastic properties by increasing the molar concentration of lipopolymers in the inner leaflet of the bilayer. For example, using wide-field single molecule microscopy we previously showed that lipopolymers act as diffusion obstacles in physisorbed polymer-tethered lipid bilayer systems. These polymer-induced obstacles result in intriguing membrane dynamics properties, such as obstacle-induced obstructed diffusion and transbilayer coupling of obstructed diffusion [15,20]. Interestingly, mean-field calculations have shown that membrane elastic properties can also be changed by altering lipopolymer molar concentration [21,22]. For instance, we recently reported that the lateral stress imparted at heightend lipopolymer concentrations result in buckle-driven delamination of a physisorbed polymer-tethered lipid bilayer [23]. Quantitative analysis of the distribution of dye-labeled probe molecules confirmed that the observed buckling phenomenon is not accompanied by phase separation of lipopolymers and lipids. Due to the reproducibility of lipopolymer concentration-specific buckling patterns, a metric between buckling structures and membrane elasticity could be derived [24]. Remarkably, in the case of poly(2-ethyl-2-oxazoline) and poly(ethylene glycol) (PEG) lipopolymers, buckling structures were found to act as lipid diffusion barriers, thus causing the compartmentalization of the polymer-tethered membrane [23]. Here bilayer compartmentalization was attributed to a stress relaxation effect in the bottom monolayer, which includes the partial penetration of polymeric chains of lipopolymers into the hydrophobic region of the buckled monolayer, thereby preventing formation of the bilayer at buckling structures (Figure 1).

In the current work, we build on our previous experiments and report the formation of physisorbed polymer-tethered lipid bilayers characterized by a stable lateral gradient in lipopolymer concentration (TYPE 1). We also present a method, in which a sharp boundary between low (no buckling) and high (buckling) lipopolymer concentration regions is obtained (TYPE 2). These surfaces represent a powerful tool to modify a single parameter in a complex, multivariate environment and to study the system’s response to changes of this single parameter. While different methods have been pursued to design polymer thin films with gradients [25] and sharp boundaries in particular polymer properties [26,27], previous related activities on solid-supported model membranes remain limited to bilayer patterning [28,29,30] and the formation of transient gradients of charged membrane constituents in a patterned bilayer using electrophoresis [28].

## 2. Results and Discussion

### 2.1. Physisorbed Polymer-Tethered Phospholipid Bilayer with Gradual Change in Lipopolymer Concentration (TYPE 1)

**Figure 2:**
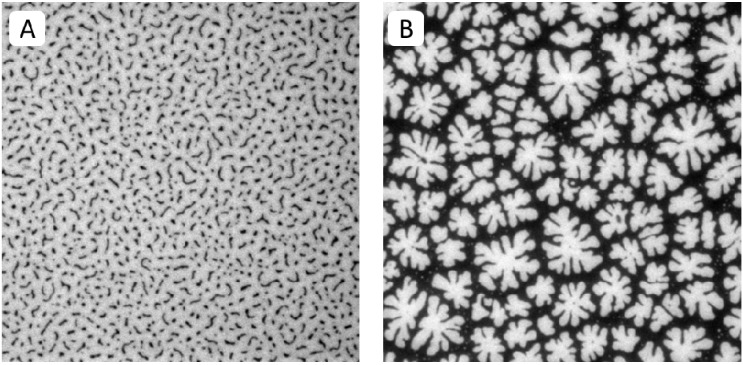
EPI micrographs of physisorbed polymer-tethered lipid bilayers of 5 mol % (**A**) and 40 mol % DSPE-PEG5000 (**B**). All pictures were taken with 40× objective and 1.6× Optovar magnification. The size of the micrographs is 100 µm × 100 µm.

Previously, we demonstrated that physisorbed polymer-tethered phospholipid bilayers with different concentrations of lipopolymers in their inner monolayer display distinct, lipopolymer concentration-specific buckling structures [23,24]. The formation of these structures was confirmed by atomic force microscopy (AFM) and was explained in terms of a stress relaxation phenomenon caused by stress-inducing lipopolymers in the membrane system. In the case of lipopolymers with amphiphilic polymer moieties, such as poly(ethylene glycol) (PEG) and poly(2-ethyl-2-oxazoline), buckling structures were easily resolvable by epifluorescence microscopy (EPI). At low lipopolymer concentrations, buckling structures were found to exist as circular or straight-sided blisters. With increasing lipopolymer concentration, blisters were reported to become more elaborate and branched and to eventually develop into a compartmentalizing buckling pattern. Figure 2A,B illustrate typical EPI micrographs of buckling structures in polymer-tethered lipid bilayers containing different amounts of DSPE-PEG5000. At 5 mol %, lateral stress is comparably low and membrane buckling regions exist as straight-sided blisters (Figure 2A). In contrast, at 40 mol %, lateral stress is high resulting in the formation of membrane-compartmentalizing buckling regions (Figure 2B).

**Figure 3:**
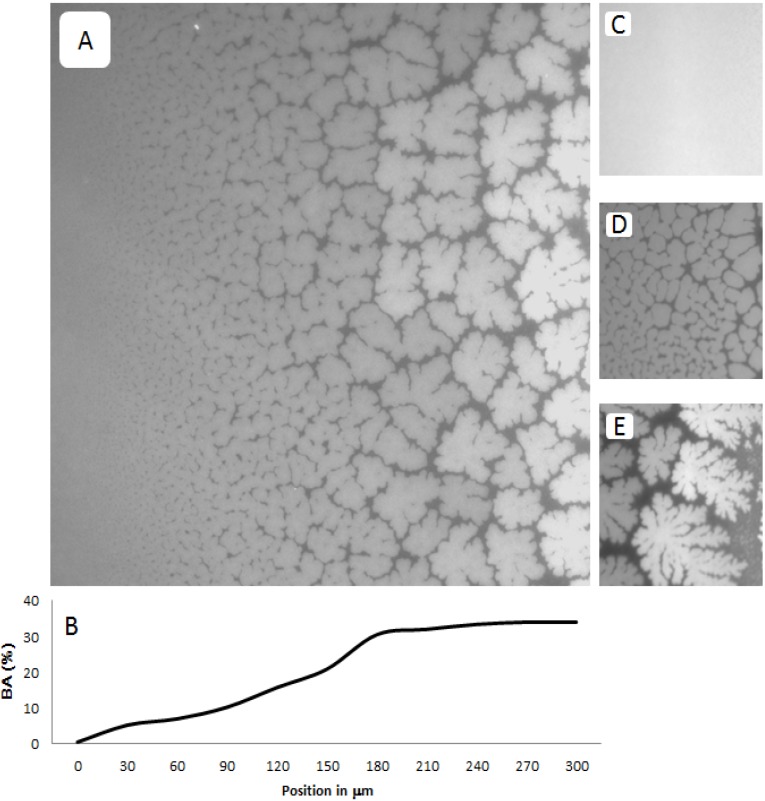
Representative EPI micrographs of a TYPE 1 physisorbed polymer-tethered lipid bilayer. The gradual change of buckling structures in Figure 2A indicates the existence of a lateral lipopolymer gradient in the membrane system (20× magnification). Figure 3B illustrates the corresponding change in bearing area, *BA* (percentage of buckled membrane region). Figure 3C–E show magnified micrographs (40× magnification) of bilayer regions characterized by differences in buckling formation: no buckling (**C**), partially branched blisters (**D**), and well developed, bilayer-compartmentalizing buckles (**E**). The image size of A is 320 µm × 320 µm, whereas that of C, D and E is 160 µm × 160 µm.

Figure 3A–E shows representative EPI micrographs obtained from a TYPE 1 polymer-tethered lipid bilayer system. As outlined in the *Experimental Section*, the lipopolymer gradient in TYPE 1 membranes was allowed to build up at the air-water interface over an incubation time period of 40 min, prior to film transfer to the solid substrate. Figure 3A presents a lower magnification micrograph captured using a 20× objective, which clearly illustrates the gradual transition from a region without optically resolvable buckling structures to one with a well-developed, bilayer-compartmentalizing buckling pattern. As the corresponding bearing area, *BA*, data (*BA* quantifies percentage of buckled membrane region) in Figure 3B illustrate, the length scale of the gradient between buckling-free regions (low lipopolymer concentrations) and those with well-developed compartmentalizing buckling structures (high lipopolymer concentrations) for this incubation time period is about 200 µm. Figure 3C–E depict higher magnification micrographs using a 40× objective of different regions of a TYPE 1 bilayer sample, which are distinct in terms of buckling formation. Figure 3C exemplifies the region without optically resolvable buckling structures, which suggests a polymer-tethered lipid bilayer with less than 5 mol % DSPE-PEG5000. The bilayer area in Figure 3D is characterized by straight-sided, partially branched blisters, indicative of a local DSPE-PEG5000 molar concentration of 5–10 mol % [24]. This micrograph also illustrates the tendency of sufficiently long buckling ridges to compartmentalize the lipid bilayer. Figure 3E shows a region of well-developed, bilayer-compartmentalizing buckling structures indicating a local DSPE-PEG5000 molar concentration of 15–20 mol %.

**Figure 4:**
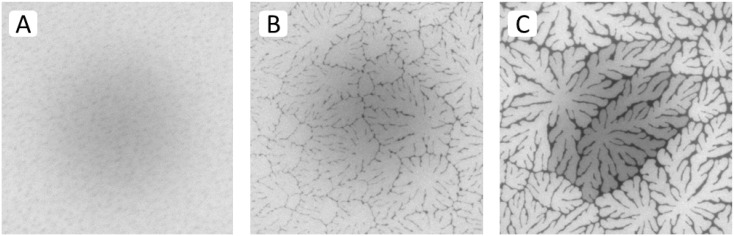
Fluorescence recovery after photobleaching of dye-labeled lipids in different regions of a TYPE 1 bilayer sample (images taken 1.5 min after spot photobleaching) exhibiting a buckling-free region (**A**); a region with branched buckling structures (**B**); and a region with bilayer-compartmentalizing buckles (**C**). The micrographs illustrate the fluidity of lipids in the bright (buckle-free) regions of the bilayer and confirm the ability of buckling structures to act as efficient lipid diffusion barriers, as reported previously [23]. (Image size: 160 µm × 160 µm).

Results from spot bleaching experiments in Figure 4A–C illustrate the influence of buckling structures on lipid lateral fluidity in different regions of a TYPE 1 bilayer. In the region without optically resolvable buckling structures (Figure 4A), the circular bleaching spot exhibits a gradual transition of the bleaching intensity indicating good fluidity within the bilayer (images taken 1.5 min after spot photobleaching). In the region of straight-sided, partially branched blisters (Figure 4B), qualitatively similar fluorescence recovery can be observed, which displays lateral fluidity outside buckling areas. Figure 4C best demonstrates that buckling areas act as efficient lipid diffusion barriers, as reported previously for physisorbed polymer-tethered lipid bilayers containing poly(2-ethyl-oxazoline) or PEG lipopolymers [23,24].

Combined AFM and spot photobleaching experiments revealed that in such cases no lipid bilayer can form on top of buckling regions [23]. Consequently, in TYPE 1 bilayers, these regions of “buckling-induced dewetting” cause the formation of diffusion obstacles at low to medium lipopolymer concentrations and the compartmentalization of the lipid bilayer system at high lipopolymer concentrations. It should be pointed out that the spot photobleaching method does not provide reliable quantitative information about lipid diffusion, as the size of the bleaching spot exceeds the average distance between buckling structures. Previously, such quantitative information was obtained using single molecule tracking experiments of dye-labeled and quantum dot-conjugated lipids in polymer-tethered membranes [15,23]. Notably, the available single molecule tracking data reveal a complex length scale-dependent lipid diffusion behavior in physisorbed polymer-tethered lipid bilayers, which exhibits remarkable parallels to those observed in plasma membranes. At sub-optical resolution length scale (~100 nm), wide-field single molecule fluorescence microscopy experiments show that lipid diffusion is well described by a model of obstacle-induced obstructed diffusion [15]. Here the degree of obstruction is determined by the density of lipopolymers in the membrane system. Interestingly, the observed obstruction of lipid diffusion at this length scale seems to be, in part, associated with a lipopolymer-induced roughening of the bilayer, which alters membrane tension [20]. At micron-size length scale, the formation of diffusion barriers in buckled regions reveals a second type of obstructed lipid diffusion. In this case, the degree of obstruction is determined by the length and connectivity of buckles. The complex lipid diffusion behavior in physisorbed polymer-tethered membranes was recently demonstrated through long-term tracking of photostable quantum dot-conjugated lipids [23]. These experiments not only showed a lipopolymer density-dependent obstruction of lipid diffusion over the entire detected length scale range, but also exhibited the feature of hop diffusion at a particular length scale (qualitatively similar to plasma membranes) [20]. It should be noted that the described lipid diffusion properties are distinct from those reported on chemisorbed polymer-tethered lipid bilayers [14].

**Figure 5:**
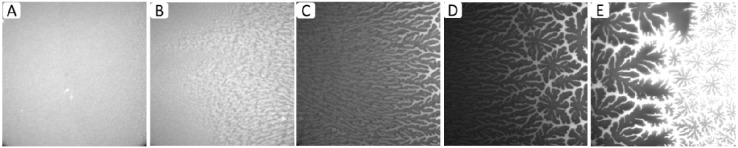
EPI fluorescence micrographs of different regions of a TYPE 1 physisorbed polymer-tethered monolayer illustrating the gradient in terms of buckling structures representative of changes in lipopolymer density: no optically resolvable buckles (**A**); straight-sided blisters (**B**); increasingly branched blisters (**C**); branched blisters and compartmentalizing buckles (**D**); and compartmentalizing buckles (**E**). Image size: 160 µm × 160 µm.

**Figure 6:**
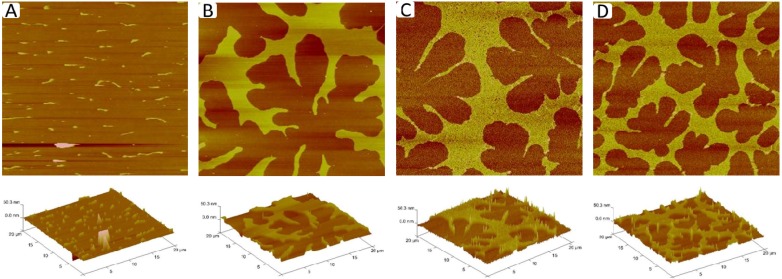
AFM micrographs of different regions of a TYPE 1 physisorbed polymer-tethered monolayer exhibiting distinct degrees of membrane buckling: straight-sided blisters (**A**); and compartmentalizing buckles of decreasing compartment size (**B**–**D**). The decreasing compartment size indicates increasing lipopolymer molar concentrations. Image size: 20 µm × 20 µm. The buckle amplitude is about 7.5 ± 1.5 Å [24].

Physisorbed polymer-tethered lipid bilayers not only show fascinating diffusion behavior, but are also characterized by interesting mechanical properties. Previously, mean-field calculations have shown that the mechanical properties of polymer-tethered membranes depend on lipopolymer density [21,22]. Interestingly, the bending elasticity, *K_c_*, of a typical red blood cell membrane of about 50*k_B_T* corresponds to that of a polymer-tethered lipid bilayer of 5 mol % DSPE-PEG5000 and *K_c_* = 400*k_B_T* of a typical membrane of Dictyostelium discoideum (wild type) is comparable to *K_c_* values in polymer-tethered membranes of 20 mol % DSPE-PEG5000 [31,32] In contrast, a fluid lipid bilayer without lipopolymers is notably softer than typical cell membranes. Importantly, there is an empirical correlation between the extent of buckling formation and membrane elastic properties. A more quantitative relationship between buckle formation and membrane elasticity can be developed by linking experimentally determined buckling parameters, such as the buckling width, *2b*, or the maximum height of buckles, *w_max_*, to mean-field calculations of polymer-lipopolymer mixtures and buckling theory of an Euler column [24]. In this case, the Euler column approximation can be applied because the buckling width is notably larger than the overall membrane thickness, *h*, and because the Young’s modulus of the glass substrate is much higher than that of the polymer-tethered membrane [33]. In the case of compartment-forming buckling structures, information about the density of lipopolymers and the corresponding membrane elasticity can be also obtained by determining the compartment density, *N_corr_* [24]. The buckling parameter information needed for quantitative correlation can be best acquired from the analysis of EPI and AFM micrographs of polymer-tethered lipid monolayers. Figure 5A-E illustrates representative EPI micrographs of different regions within a TYPE 1 polymer-tethered monolayer sample. The micrographs depict the gradual transition from regions of low lipopolymer concentration (≤5 mol % DSPE-PEG5000) (Figure 5A) to those of elevated lipopolymer concentration (~30 mol % DSPE-PEG5000) (Figure 5E). Monolayer micrographs show typical phase inversion (relative to corresponding bilayer system) observed on polymer-tethered membranes with PEG lipopolymers (*i.e.*, bright phase represents buckling regions in monolayer , while dark phase represents buckling regions in bilayer) [24]. Figure 6A–D displays corresponding AFM micrographs of different regions of a typical TYPE 1 monolayer (length scale 20 µm × 20 µm). While Figure 6A shows a monolayer region, which is characterized by straight-sided blisters, Figure 6B–D depict a compartmentalizing buckling pattern of decreasing compartment size. Using previously applied protocols [23], analysis of buckling width, *2b* (Figure 6A), and compartment density, *N_corr_* (Figure 6B–D), suggests lipopolymer molar concentrations of 4 mol % (Figure 6A), 16 mol % (Figure 6B), 31 mol % (Figure 6C), and 36 mol % (Figure 6D), associated with a change in the plane strain modulus, $E_{f}^{*}$, of the membrane from 1.9 to 7.3 MPa.

The data presented for TYPE 1 membranes in Figure 3, Figure 4, Figure 5 and Figure 6 bring to light a fascinating model membrane system with gradually changing properties of membrane organization, dynamics, and elasticity. Notably, the length scale of the gradient in TYPE 1 membranes depends on the incubation time after addition of the POPC solution to the mixed POPC-DSPE-PEG5000 monolayer at the air-water interface. Longer incubation times lead to increasing length scales of gradients between buckling-free regions (low lipopolymer concentrations) and those with compartmentalizing buckling structures (high lipopolymer concentrations), due to diffusional relaxation processes of lipids and lipopolymers at the air-water interface. The significance of the TYPE 1 architecture is that gradients become static and do not change over time after LB transfer. This static behavior is caused by the physisorption of lipopolymers onto the glass substrate preventing the gradual relaxation of the lipopolymer gradient. Resulting differences in lipopolymer density in TYPE 1 systems demonstrate the ability to maintain regions of different lateral stress within one membrane sample. These regions manifest themselves in terms of clearly distinguishable buckling structures. Such buckling structures represent a buckle delamination of the membrane seen as stress relaxation phenomena. Furthermore, the lateral lipopolymer gradient leads to remarkable length scale-dependent lipid fluidity in TYPE 1 bilayer systems ranging from regions of low obstruction of lipid diffusion to those characterized by significant lipopolymer-induced obstructed and hop diffusion processes. Here it is important to recognize that the physisorption of lipopolymers on the glass substrate does cause the obstruction of lipid diffusion, but typically not to the degree of complete membrane immobilization. A simple fluid lipid bilayer system with a comparable static gradient does not appear to be feasible as the lateral mobility of lipids will decrease any previously formed gradient over time. This is beautifully illustrated by the analysis of transient gradients of charged lipids in micropatterned solid-supported lipid bilayers [28]. In this case, the gradient of charged, dye-labeled lipids was created by applying an electric field and the time evolution of the gradient was analyzed after turning off the applied electric field, thus providing information about lipid diffusivity. However, in the case of engineered solid substrates with specific gradient properties (e.g., surface charge or curvature), lipid bilayer structures with membrane constituent gradients seem possible. An alternative gradient strategy could be the usage of polymerizable lipids to build a lipid bilayer system with a lateral gradient in lipid crosslinking density.

### 2.2. Physisorbed Polymer-Tethered Phospholipid Bilayer with Sharp Boundary between Regions of Low and High Lipopolymer Concentrations (TYPE 2)

**Figure 7:**
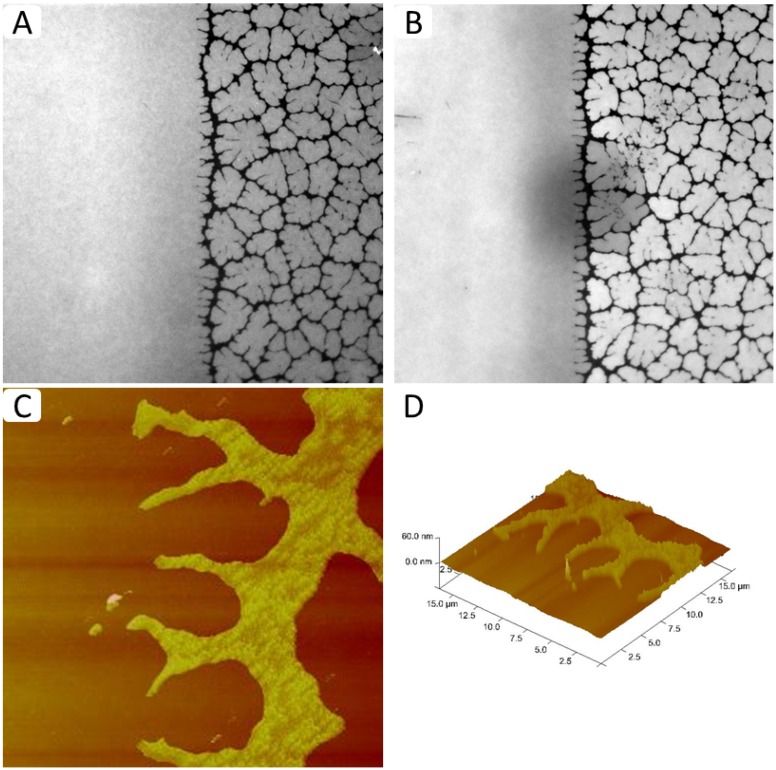
EPI (**A**,**B**) and AFM micrographs (**C**,**D**) of TYPE 2 physisorbed polymer-tethered lipid bilayer and monolayer, respectively. Micrographs confirm the existence of a sharp boundary between regions of low and high lipopolymer densities with distinct properties of membrane dynamics and elasticity. (EPI micrograph image size: 160 µm × 160 µm; AFM micrograph image size: 20 µm × 20 µm. The buckle amplitude is about 7.5 ± 1.5 Å [24].

The immobilization of physisorbed lipopolymers on the glass surface not only offers the possibility to fabricate membrane systems with lateral lipopolymer density gradients, but also those with a sharp boundary between regions of low and high lipopolymer molar concentrations. As described in the *Experimental Section*, TYPE 2 membranes were built by regulating the phospholipid-lipopolymer mixing ratio at the air-water interface and by conducting partial LB transfers at altered lipopolymer concentrations. Figure 7A–D shows representative EPI and AFM micrographs of such a physisorbed polymer-tethered lipid membrane. The EPI micrograph in Figure 7A illustrates two sharply separated membrane regions, a homogeneous region and a region characterized by compartmentalizing buckling structures. As outlined in the Materials and Methods section, the homogeneous and non-homogeneous buckled regions contain approximately 5 and 30 mol % DSPE-PEG5000, respectively. The shape of the bleaching spot in Figure 7B demonstrates the good bilayer fluidity in the homogeneous region of the membrane with the low lipopolymer density. In contrast, the partially recovered bleaching spot in the non-homogeneous region shows that the “dark phase” acts as a lipid diffusion barrier. This behavior suggests that the non-homogeneous region is not caused by phospholipid-lipopolymer phase separation, but instead is a typical fingerprint of membrane buckling and buckling-induced “dewetting” [24]. Indeed, the presence of buckling structures is confirmed by AFM micrographs in Figure 7C,D that show representative AFM data from the boundary region of a typical TYPE 2 polymer-tethered monolayer. Again it should be emphasized that the sharp boundary between regions of low and high lipopolymer densities, which exhibit distinctly different dynamic end elastic properties, remains unchanged over an extended period of time. Of course, the concept of TYPE 2 membranes should not remain limited to those with one sharp boundary. Modifications to the membrane fabrication process can be envisioned, which lead to well-defined patterned polymer-tethered bilayer systems. Previously, several successful strategies have been pursued to build patterned solid-supported lipid bilayers. For example, Groves *et al.* used patterned grids of photoresist, aluminum oxide, or gold on oxidized silicon substrates to form patterned solid-supported lipid bilayers [28]. Other patterning strategies include the photochemical patterning [29] and patterning via the controlled crosslinking of polymerizable lipids [30]. An interesting example of patterning in polymer-supported membranes represents the controlled formation of stripe phases in polymer-tethered lipid bilayers comprised of lipids and lipopolymers, in which stripe formation was controlled through changing LB transfer conditions [34].

## 3. Experimental Section

The lipopolymer 1,2-distearoyl-*sn*-glycero-3-phosphoethanolamine-*N*-[folate(polyethylene glycol)-5000] (DSPE-PEG5000) and the phospholipid 1-palmitoyl-2-oleoyl-*sn*-glycero-3-phosphocholine (POPC) were purchased from Avanti Polar Lipids (Alabaster, AL). The fluorescently labeled phospholipids Texas Red^®^ 1,2-dihexadecanoyl-*sn*-glycero-3-phosphoethanolamine triethylammonium salt (Texas Red^®^ DHPE) and *N*-(6-tetramethylrhodaminethiocarbamoyl)-1,2-dihexadecanoyl-*sn*-glycero-3-phosphoethanolamine triethylammonium salt (TRITC-DHPE) were obtained from Invitrogen Life Technology/Molecular Probes (Eugene OR). HPLC grade chloroform (Fisher Scientific, Pittsburgh, PA) was used to make mixtures of lipopolymers, phospholipids, and dye-labeled lipids prior to spreading at the air-water interface. Milli-Q water (pH = 5.5, 18 MΩ-cm resistivity; Milli-Q, Millipore, Billerica, MA) was employed as the subphase material for all experiments. Glass coverslips (24 mm × 40 mm, No. 1) purchased from VWR Scientific Products (West Chester, PA) were baked in a furnace at 515 °C for 1 h. After baking, the coverslips were cleaned using a series of sonication steps, which include 1% SDS solution (30 min), NaOH Methanol (30 min), and 0.1% HCl solution for 30 min. The coverslips were rinsed with Milli-Q water for 5 min between sonication steps and after completion of the sonication treatment. All chemicals and buffers used for the cleaning were purchased from Thermo Fisher Scientific.

### 3.1. Design of Polymer-Tethered Phospholipid Monolayer and Bilayer

Physisorbed polymer-tethered phospholipid bilayers with variations in lipopolymer concentration were built using the Langmuir-Blodgett (LB)/Langmuir Schaefer (LS) method, thereby modifying previously reported procedures [24]. Two types of membrane designs were pursued, a polymer-tethered lipid bilayer with a gradual change in lipopolymer concentration (TYPE 1) and a membrane system with a sharp boundary between low and high lipopolymer concentration regions (TYPE 2). To build TYPE 1 membrane systems, 50–60 µL of a chloroform solution of POPC and 30 mol % DSPE-PEG5000 (1 mg/mL) was first spread at the air-water interface of a film balance with a dipper (Labcon, Darlington, UK), compressed to a film pressure of 30 mN/m and allowed to equilibrate for approximately 30 mins to ensure the homogeneous spreading of lipids and lipopolymers. Next the available monolayer area was expanded to a film pressure of 25 mN/m. At this time, 30–32 µL of a 2.5 mg/mL POPC chloroform solution was added to the existing mixed POPC-DSPE-PEG2000 monolayer by equally distributing the solution along a parallel line approximately 1–2 cm from the film balance barrier (trough dimension: 10 cm × 60 cm). The Langmuir monolayer was then allowed to equilibrate for approximately 40mins at 30mN/m. During this equilibration step, a lipopolymer gradient was allowed to build up based on the lateral mobility of lipids and lipopolymers at the air-water interface. The Langmuir monolayer with the lipopolymer gradient was transferred to a glass slide using LB deposition. Resulting LB monolayers were imaged by epifluorescence microscopy (EPI) and atomic force microscopy (AFM) without further modification. To enable EPI experiments, all stock solutions used in the fabrication of LB monolayers contained 0.5 mol % of the dye labeled lipid Texas Red^®^ DHPE or TRITC-DHPE.

To fabricate TYPE 2 membrane architectures, a chloroform solution of POPC and 5 mol % DSPE-PEG5000 (1 mg/mL) was first distributed at the air water interface and compressed to 30mN/m; the area of the trough was noted at this time. After equilibrating for approximately 30 min, the film was then transferred to half of the glass slide; the area of the trough following the transfer was noted. The quantity of lipids transferred to the slide was then determined based on the difference in trough area subsequent to the film transfer. With this information, the moles of lipids remaining in the trough could be determined. This allowed the calculation of DSPE-PEG5000 required to increase the remaining lipid/lipopolymer mixture to a concentration of 30 mol % DSPE-PEG5000. A chloroform solution of DSPE-PEG5000 (5 mg/mL) was then evenly spread at the air water interface; manually stirring aided in facilitating an even concentration of lipopolymer across the film. The resulting mixture was allowed to equilibrate for approximately 30min and was then transferred to the other half of the glass slide.

In both TYPE 1 and 2 systems, polymer-tethered lipid bilayers were completed through the addition of a second monolayer with a composition of POPC and 0.5 mol % Texas Red^®^ DHPE. This monolayer was prepared through an LB approach and was added to TYPE 1 and 2 monolayers through LS transfer. EPI microscopy and FRAP were performed on the resulting bilayers with no further modifications.

### 3.2. Epifluorescence Microscopy and Analysis

Fluorescence microscopy (EPI) and spot photobleaching experiments were performed using a Zeiss Axiovert 200M (Zeiss, Oberkochen, Germany) equipped with an AxioCam MRm monochrome digital camera, as reported previously [23]. EPI micrographs were taken using a (Zeiss EC-Plan Neofluar 20×, NA = 0.5 or a Zeiss C-Apochromat, water immersion, 40×, NA = 1.2). The microscope is equipped with an additional 1.6× Optovar magnification system. EPI micrographs were analyzed using Axiovision software (Zeiss, Oberkochen, Germany) and ImageJ (National Institutes of Health) [35]. The analyzed EPI data were also compared with the results obtained using AFM. To quantify the gradual change in buckling formation along the lipopolymer gradient in TYPE1bilayers, the bearing area, BA (percentage of buckled membrane region) was systematically analyzed along the gradient using Axiovision LE software, as reported previously [24]. Here average BA values at a particular *x*-position of the EPI micrograph were obtained by averaging BA data from five micrograph segments of 15 µm × 15 µm at that *x*-position and by repeating this procedure in *x*-direction with an increment of 15 µm.

### 3.3. Atomic Force Microscopy Analysis

A Digital Instruments BioScope (Digital Instruments/Veeco Metrology Group, Plainview NY) was used to conduct atomic force microscopy (AFM) experiments on polymer-tethered lipid monolayers. Each sample was scanned using a stiff (*k* = 40 Nm^−1^) non-conductive silicon nitride cantilever (Tap 300-G, Budgetsensor/Innovative Solutions Bulgaria Ltd. Bulgaria). Regions of 20 µm × 20 µm were imaged at a resolution of 256 × 256 pixels using scan rates of 0.1–1 Hz. The monolayer samples containing the gradient structure were analyzed in air with 24 h of preparation using tapping mode. By adapting procedures reported previously [24], AFM micrographs exhibiting straight-sided blisters were analyzed in terms of buckle width, *2b*, whereas those showing compartmentalizing buckling patterns were characterized by determining compartment density, *N_corr_*, using Nanoscope IV and Nanoscope Analysis software (Digital Instruments/Veeco Metrology Group, Plainview NY). To achieve statistical significance, *N_corr_* values were also acquired using EPI. Next, the acquired values for *b* and *N_corr_* were utilized to derive the specific lipopolymer densities and plain-strain moduli of the membrane, $E_{f}^{*}$.

## 4. Conclusions

In the current work we report the fabrication of two types of physisorbed polymer-tethered lipid bilayers where the lateral distribution of lipopolymers can be regulated. In the case of TYPE 1 membrane systems, a gradient in lipopolymer concentration is achieved by adjusting the phospholipid-lipopolymer mixing ratio at the air-water interface prior to LB transfer. Importantly, this gradient becomes static after the transfer of the polymer-tethered membrane to the solid (glass) substrate. TYPE 1 polymer-tethered lipid bilayers have exciting properties including gradual changes in length scale-dependent lipid diffusivity and membrane elasticity. In contrast, TYPE 2 polymer-tethered membranes are characterized by a sharp boundary between regions of low (no buckling structures) and high (with buckling structures) lipopolymer concentrations. Again the sharp boundary remains static after physisorption of the polymer-tethered membrane to the solid substrate. Our work is significant as it demonstrates the ability to create biomembrane-mimicking membrane architectures with stable gradients in membrane composition affecting dynamics and mechanical properties. Such membrane systems are potentially useful to investigate the influence of membrane packing and compartmentalization on the lateral diffusivity of membrane proteins. Furthermore, they could be employed as biomembrane-mimicking cell substrates to characterize properties of plated cells, similar to previously reported experiments using solid-supported lipid bilayers [3,4].
